# Institutional Housekeeping

**Published:** 1911-10-21

**Authors:** 


### INSTITUTIONAL HOUSEKEEPING.

(Contributions invited: Workers' Questions will be welcome.)

### Housekeeping—A Science and an Art.

Housekeeping as a science demands an exact training which has only recently been opened up to women. Perhaps the nearest approach to this training hitherto obtainable has been that of the steward of large establishments, including private houses, barracks, hotels, Poor Law institutions or hospitals. Training in the sense of formal initiation into their career few stewards obtain. It is rather a process of gradual appropriation of knowledge floating about, so to speak. But for these posts there has usually been a preparation extending over some years of exclusive attention to the duties connected with household management on a large scale, and in this respect the training of a steward for his domestic responsibilities differs from that of the matron, who during the period when she has been acquiring the like knowledge has usually been laden with duties of a more urgent nature, and has been unable to devote her energies exclusively to the task of mastering the principles of sound housekeeping.

### A New Science.

There is an immense deal to learn in the new science of housekeeping. It comprises a knowledge of foodstuffs, with their chemical constituents, their food value, their market price, their varying grades of quality, the forms of adulteration against which it is necessary to guard, the best processes of cooking, and the alterations produced by cooking processes. It includes a knowledge of the processes connected with the cleaning of structures, of furniture and equipment, of utensils and of clothes, together with the principles of aseptics and acquaintance with the properties of substances used in disinfection and in cleaning. It involves a knowledge of the right methods of securing efficient service, of a just scale of wages, and of the adjustment of the household staff to the size of the establishment, so that there may be neither the confusion of under- nor the wastefulness of over-staffing. It is concerned with all the linen, hangings, carpets, and uniforms in use, and demands a knowledge of their fabric no less than of the mode of making them up for use. Household accounts are included in its scope, with an examination of the modes which have borne the test of experience in preventing waste and checking expenditure.

### The Practical Housekeeper.

None of these branches of knowledge can safely be ignored by the practical housekeeper, but in many she is terribly hampered whenever she attempts to look beneath the surface. She is cheated, and knows that she is cheated, by those who provide her with every necessary. Too often she concentrates all her energies on skimping those she has to feed and house, saving pennies in unpopular economies while pounds are running away unperceived without purpose. She is unsuccessful because she does not know, and she does not know because she has had no facilities for learning.

The art of housekeeping is different from its science. The woman who treats her housekeeping as an art may be lacking in knowledge, and probably in this case takes four times the amount of trouble about everything than is necessary, but she at least produces an effect. She aims at something definite, whether economy, or display, or quality, or solid comfort, and she subordinates all her endeavours to that end. When, by a rare combination of qualities, the scientific housekeeper likewise understands and practises household management as a beautiful art, life takes on quite a new complexion for her family, her guests and dependents. There is no need to describe her. It has been done too well already, once for all, by Solomon.

Every day the knowledge of housekeeping is becoming more important, but it is still inaccessible to many who would fain be learners. Every advance of practical domestic science makes the art more agreeable to practise. It is a growing science, and as such needs observation and continual study from those who desire to excel. We propose to furnish our readers with material for increasing their own knowledge, and informing themselves on many points, both practical and theoretical, needing elucidation in the upkeep of institutions large and small. Queries on household subjects are invited, and will receive the attention of experts. And a sample department will deal with the price and quality of articles in common use on which an expert opinion may be desired.

### The Price of Provisions in London.

Should any of our readers be disposed to think that the last word on scientific housekeeping has been said in the for the most part admirably administered hospitals included within the scope of King Edward's Hospital Fund, we append the table showing the highest and lowest of the prices which were being paid on December 31, 1910, for provisions in 105 hospitals situated within greater London:—

| Description | Highest price £ | s. | d. | Lowest price £ | s. | d. |
|---|---|---|---|---|---|---|
| Topsides of beef, per lb. ... ... | 0 | 0 | 10 | 0 | 0 | 3 |
| Legs of mutton, per lb. ... ... | 0 | 0 | 9½ | 0 | 0 | 4 |
| Fish, per lb. ... ... ... ... | 0 | 0 | 6 | 0 | 0 | 1½ |
| Fowls, each ... ... ... ... | 0 | 4 | 0 | 0 | 1 | 9 |
| Bacon, per lb. ... ... ... | 0 | 1 | 0 | 0 | 0 | 5½ |
| Butter, per lb. ... ... ... | 0 | 1 | 2 | 0 | 0 | 10½ |
| Eggs, new laid, per 120 ... ... | 1 | 0 | 0 | 0 | 8 | 6 |
| Eggs, cooking, per 120 ... ... | 0 | 14 | 6 | 0 | 7 | 6 |
| Flour, per sack, 280 lb. ... ... | 2 | 6 | 8 | 1 | 5 | 6¼ |
| Milk, per gallon ... ... ... | 0 | 1 | 10 | 0 | 0 | 7¾ |
| Bread, per 4 lb. ... ... ... | 0 | 0 | 8 | 0 | 0 | 4 |
| Tea, per lb. ... ... ... ... | 0 | 1 | 8 | 0 | 0 | 11½ |
| Sugar, per cwt. ... ... ... | 1 | 4 | 6 | 0 | 10 | 0 |
| Rice, per cwt. ... ... ... | 1 | 2 | 0 | 0 | 7 | 0 |
| Potatoes, per cwt. ... ... ... | 0 | 9 | 0 | 0 | 3 | 3 |

